# Towards the World’s Smallest Gravimetric Particulate Matter Sensor: A Miniaturized Virtual Impactor with a Folded Design

**DOI:** 10.3390/s22051727

**Published:** 2022-02-23

**Authors:** Navpreet Singh, Mohannad Y. Elsayed, Mourad N. El-Gamal

**Affiliations:** 1 Electrical and Computer Engineering, McGill University, Montreal, QC H3A 0G4, Canada; mourad.el-gamal@mcgill.ca; 2 MEMS Vision International Inc., Montreal, QC H4P 2R9, Canada; mohannad.elsayed@mems-vision.com

**Keywords:** MEMS, sensors, particulate matter, PM2.5, 3D printing, virtual impactor

## Abstract

The increasing air pollution across the globe has given rise to a global health crisis that is increasing at an alarming rate. Every year, millions of people lose their lives due to health risks caused by air pollutants. Hence, there is a pressing need for better solutions to accurately measure the amount of air pollution. This work is aimed at designing a highly compact, accurate, low-cost, self-resettable, and easy-to-use gravimetric-based particulate matter sensor solution for portable applications. Previous attempts have failed to realize true miniaturization, due to the size constraints of the *virtual impactor* needed—a mechanism that segregates the particulate matters based on their sizes. Our complete particulate matter sensor solution consists of three components (i) a piezoelectric resonating membrane, (ii) a virtual impactor, and (iii) a thermophoretic mechanism to reset the sensor. This paper presents a novel design of the virtual impactor, based on a folded configuration. This helps realize the entire system in a volume of 20 mm × 20 mm × 10 mm. We report here the design, working principles, fabrication, and experimental results of the virtual impactor.

## 1. Introduction

Air quality around the globe has been steadily declining since the rise of the manufacturing industry, catering to ever-increasing human needs [1]. This air quality is ascertained by the amount of pollutants in the air. Both the type and the amount of the pollutants play a major role in the air quality of a region. Other factors that affect the air quality include weather conditions such as wind speed, temperature, and precipitation.

Air pollution is a mixture of hazardous human-made and natural substances. Primary sources of air pollution include vehicular emissions, fuel oils, by-products of manufacturing and power generation units, etc., and these are all human-made sources. Natural sources include smoke from wildfires, gases from volcanic eruptions, etc. Some of these air pollutants are poisonous, and inhaling them can cause serious health problems. Air pollution kills nearly 4.2 million people globally every year [1]. Nations with the majority of their population in the low- and middle-income households are at the highest risk; this includes nations from Asia, Africa, and the Middle East.

A major portion of air pollution can be attributed to “*particulate matter*”. Particulate matter (PM) is a mixture of solid particles and liquid droplets found in the air, such as dust, soot, dirt, or smoke. Based on the sizes of these particles, particulate matter can be divided into two main categories:

- PM_10_: inhalable particles, with aerodynamic diameters of 10 μm and smaller;
- PM_2.5_: fine inhalable particles, with aerodynamic diameters that are generally 2.5 μm and smaller.

Particles with sizes of less than 10 μm can travel deep into the lungs and can even enter the bloodstream. Ultrafine particles with sizes smaller than 2.5 microns may penetrate the human skin and cause various serious health issues. The WHO warns against the health risks that could arise from any amount of particulate matter, however small. While no lower limits have been recognized, the aim should be to keep the concentration of the particulate matter as low as possible. The WHO has set guideline limits to achieve the lowest concentration of particulate matter, and these guideline limits are as follows [2,3]:
PM2.5:  10 µg/m^3^ annual mean
25 µg/m^3^ 24-h mean PM10:20 µg/m^3^ annual mean
50 µg/m^3^ 24-h mean

These guidelines necessitate the need for developing new solutions to accurately measure the amount of particulate matter present in the air. The existing solutions are broadly classified into two categories. The first category is based on directly measuring the mass of the particulate matter present in the air and is termed the “*gravimetric method*”. The particles are collected on a filter over a certain period and are then weighed in a laboratory at the end of this fixed period. Although this method is the gold standard of measuring the amount of particulate matter, these solutions do not provide real-time sensing. A filter needs to be taken to a laboratory for analysis, which causes delays to every measurement. Additionally, equipment based on these methods is relatively expensive, and their size limits their widespread use [4]. It is inefficient to deploy multiple sensing systems of this type in a city for example, while collecting data from only limited locations around that city [5,6,7].

Another category of monitors is based on the principle of *light scattering*. The particles are attracted inside a small chamber (e.g., using an air fan), then a laser beam operating at a given wavelength is directed inside the chamber. The system then measures the amount of laser energy scattered back by the particles. The amount of scattering can give an estimate of the amount of particles in the enclosed chamber. Unlike gravimetric solutions that directly measure the ***weight*** of the particles in the air, these methods only report the ***number*** of particles present in the air. Since the WHO guidelines are based on the weight of the particles, light-scattering-based monitors must convert the number of particles to weight of particles by estimating the size distribution of the particles and assuming a fixed density for all of the measured particles. This often leads to inaccurate results [8]. However, these monitors are the best solutions in the cases where the number of particles needs to be calculated, such as in the cleanroom of a semiconductors research facility. These methods remain relatively expensive (e.g., priced in the range of hundreds of US dollars).

The different limitations discussed above clearly necessitate the need for new solutions to overcome all of these shortcomings, while consistently providing highly accurate measurements. Recent advances in MEMS technologies have encouraged the use of resonators to measure the amount of gases or particulate matter present in the atmosphere. Resonating structures such as cantilevers [9], surface acoustic wave resonators (SAWR) [10], and capacitive micromachined ultrasonic transducers (CMUT) [11] can all be used as microscopic weighing scales which, on the deposition of masses on their sensing areas, can register shifts in the resonant frequency or the phase of a signal. Such solutions proved successful in measuring the amount of matter in the air but failed to reset themselves for another measurement. Therefore, most of these solutions provide single-use measurements and need to be manually cleaned/reset after each measurement.

Our work targets the development of the world’s smallest, standalone, real-time, gravimetric-based, particulate-matter-sensing solution. It is based on using a piezoelectric resonator manufactured in a multi-user MEMS process (as reported in [12]). The solution consists of three components: (i) a 3D-printed channel which directs the flow of incoming particulate matter towards the sensing resonator; (ii) A resonator that shifts its resonance frequency based on the weight of the particulate matter deposited on it; then, (iii) a mechanism that clears the particles off the resonator. This mechanism could use the process of thermophoresis or di-electrophoresis. 

This paper focuses on the folded implementation of the virtual impactor, which is the mechanism that segregates and directs the particles with the sizes of interest towards the sensing region. The paper begins with a brief introduction of the overall system and of the sensing mechanism; then, the design and characterization of the virtual impactor are presented in detail.

## 2. System Overview

The complete particulate matter sensing solution can be envisioned as a three-part system [10]:

1. A mass sensing unit;
2. A virtual impactor;
3. A thermophoretic reset mechanism.

**Figure 1:**
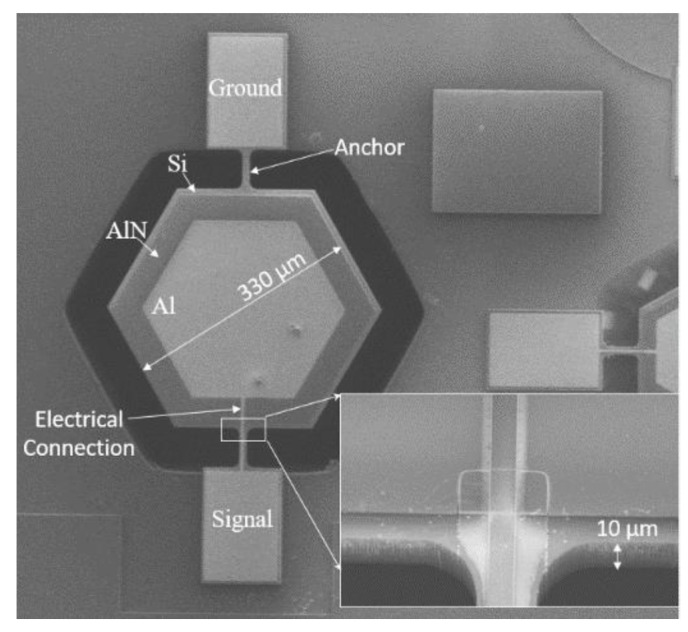
SEM of the piezoelectric resonator [12].

A piezoelectric resonator that registers a shift in its resonant frequency on the deposition of mass on its sensing membrane is the core sensing unit of the system. This was successfully demonstrated and reported in [13] and is shown in Figure 1. The resonator was fabricated using silicon as the resonating membrane, aluminum nitride as the piezoelectric material, and aluminum as the top electrode. The silicon membrane is hexagonal with a diameter of 330 μm and a thickness of 10 μm.

**Figure 2:**
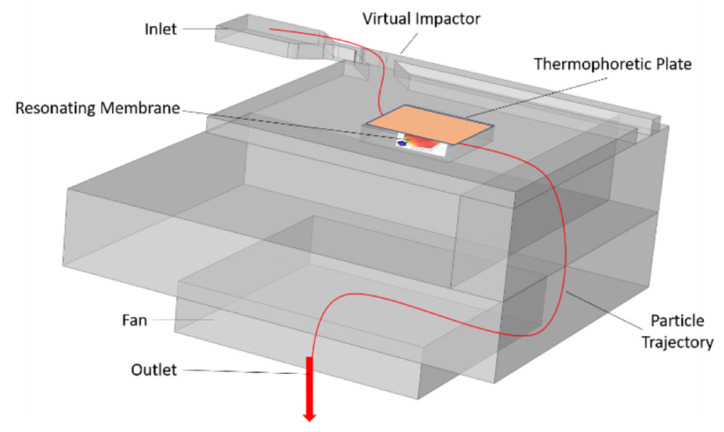
The complete solution for PM sensing (red line shows the trajectory of the particle inside the sensor).

The focus of this paper is a virtual impactor consisting of a set of channels that help segregate the particles based on their aerodynamic size, then direct them towards the sensing unit. Figure 2 shows the trajectory of the particles (red curved line) entering from the inlet, being segregated by the virtual impactor, and then being directed towards the resonating membrane. The particles then leave the sensor via the exit, and the membrane is reset using the thermophoretic system (in orange color).

## 3. Miniaturized Virtual Impactor

### 3.1. Working Principle and Design Parameters

The need to segregate the particulate matter based on their aerodynamic sizes arises from the WHO guidelines. The size of the particulate matter particles largely determines the extent of the damage caused to the environment and human health. Therefore, the particulate matter must be measured separately in each size categories [14].

**Figure 3:**
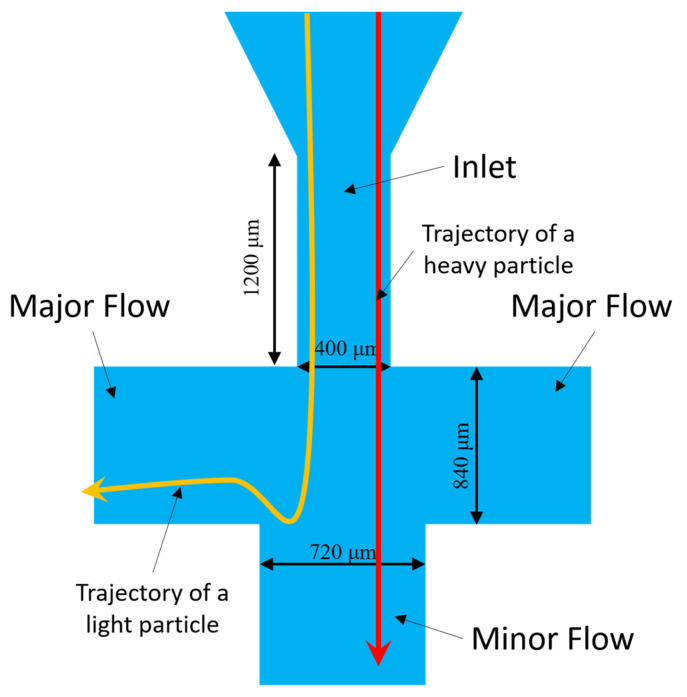
Virtual impactor with dimensions, showing the major and minor flow channels. The red line depicts the trajectory of a heavier particle, and the yellow line depicts the trajectory of a lighter particle.

A virtual impactor is a device that is used to separate the particles by aerodynamic size into two airstreams [15]. The working principle is like a conventional impactor, but with a small modification: instead of using a surface for impact, it is replaced with a virtual space of slow-moving or almost stagnant air. Larger particles, instead of colliding with the impaction surface, are then collected in a minor flow channel, and the smaller particles are directed towards the major flow channels, as shown in Figure 3. 

Particles passing through the accelerating nozzle are directed towards the minor flow channel. As the particles are advancing towards the minor flow channels, they encounter a diverting flow (at 90 degrees) away from the minor flow channels. This flow is termed as major flow, and this is where the particle size separation occurs. Smaller particles, with lower inertia, tend to follow the streamlines formed by the major flow (yellow line in Figure 3), whereas larger particles with a higher inertia tend to not diverge from the path and end up in the minor flow channel (red line in Figure 3). The ratio of the major and minor flows and the physical dimensions of the inlet nozzle and the major and minor flow channels are the critical parameters that set the efficiency of the size-based separation. 

The virtual impactor utilizes the inertial force of the particles in the air flow to separate them into two different channels. Flow rates become important parameters when designing a virtual impactor. The particle cut-off diameter of a virtual impactor is then defined as the threshold diameter at which particles are separated into two different streams. The 50% cut-off particle diameter of the virtual impactor (*D_p_*50) is defined as the geometrical diameter of the particle corresponding to a 50% collection efficiency. The particles in the air have different size irregularities. Hence, an aerodynamic *equivalent diameter* is used as the accepted metric for calculating the efficiency of a virtual impactor. The dependence of the dimensions of the virtual impactor on the cut-off diameter is governed by Stoke’s law [16]. The cut-off characteristics of the virtual impactor can be expressed in terms of the Stokes number as:

(1) $$Stk=\frac{\rho_{p}UD_{p}{}^{2}C_{c}}{18µW}\;,$$

where *Stk* is the Stokes number, ρ*_p_* is the density of the particles, *U* is the average air velocity, *W* is the width of the inlet slot of the virtual impactor, *D_p_* is the aerodynamic diameter of the particles, *µ* is the viscosity of the gas that carries the particles. The viscosity of the carrier gas is a function of temperature and can be evaluated as [17]:

(2) $$µ={µ}_{0}{\left(\frac{T}{T_{0}}\right)}^{\frac{1}{2}}\frac{\left(1+\frac{110}{T_{0}}\right)}{\left(1+\frac{110}{T}\right)}\;,$$

where *µ*_0_ is the viscosity of air at *T*_0_ = 296.2 K. *C_c_* is the Cunningham correction factor, which is responsible for the non-continuum Stoke’s drag on the particles. The Cunningham factor can be approximated by [18]:

(3) $$C_{c}=1+1.657K_{n}\;,$$

where *K_n_ = 2λ/D_p_* is the particle’s Knudsen number, and *λ* is the mean free path of the air which is a function of temperature and pressure as [16]:

(4) $$\lambda \;\left(µm\right)=3.023\times 10^{-4}\frac{T}{P\left(1+\frac{110}{T}\right)},$$

where *T* is the temperature and *P* is the atmospheric pressure.

Another important parameter governing the flow field in a virtual impactor is the Reynolds number, which is defined as the ratio of the inertial force to the frictional force and can be calculated as:

(5) $$Re=\frac{\rho WU}{µ}$$

where ρ is the density of the carrier gas. 

The Stoke’s number is therefore based on the pressure, temperature, and actual fluid velocity at the exit of the inlet of the virtual impactor (Figure 3). The dimensionless parameter ${\sqrt{Stk}}_{50}$ determines the particle separation, and it corresponds to the 50% cut-off particle diameter, *D_p_50*. The particle density ρ_p_ is set to 1 gm/cm^3^ according to the specifications of the test particles used in testing. Based on previous work [15,16,17,18], the cut-point stokes number ${\sqrt{Stk}}_{50}$ is set to 0.23 in our design. A virtual impactor with this Stoke’s number results in 50% of the particles with the cut-off diameter being collected by the major flow channel of the virtual impactor. Equation (1) relates different physical parameters of the virtual impactor with the Stoke’s number. Since our virtual impactor aims to segregate particles smaller than 2.5 µm into the major flow channel, the *D_p_* is set to 2.5 µm. With these fixed values for the *Stk* and *D_p_*, other parameters can be easily calculated using Equations (1)–(4). The Knudsen number, calculated using Equation (4), is dependent on the temperature and atmospheric pressure and helps to determine the Cunningham number using Equation (3). According to Equation (1), the only parameters left to be finalized are the inlet width *W* and the air velocity *U*. The Reynolds number, calculated using Equation (5), must be in the range of 500 and 3000 for a reasonable collection efficiency. Using Equations (1) and (5), we can then find values for *W* and *U*. Another factor influencing the value of *U* is the flow-rate specification of the external fan used to maintain the air flow inside the channels of the virtual impactor. The average air velocity *U* can also be calculated by performing finite element simulations and was determined to be about 1000 m/s in our design, which corresponds to an air flow rate of about 16 L/min, based on the dimensions of the inlet channel. This flow is maintained by an external fan with a specified flow rate of 16.9 L/min. Using these conditions, the inlet channel width *W*, and hence the width of the major flow channel, were calculated to be 400 µm and 840 µm, respectively [19,20,21,22].

### 3.2. Design and Simulations

**Figure 4:**
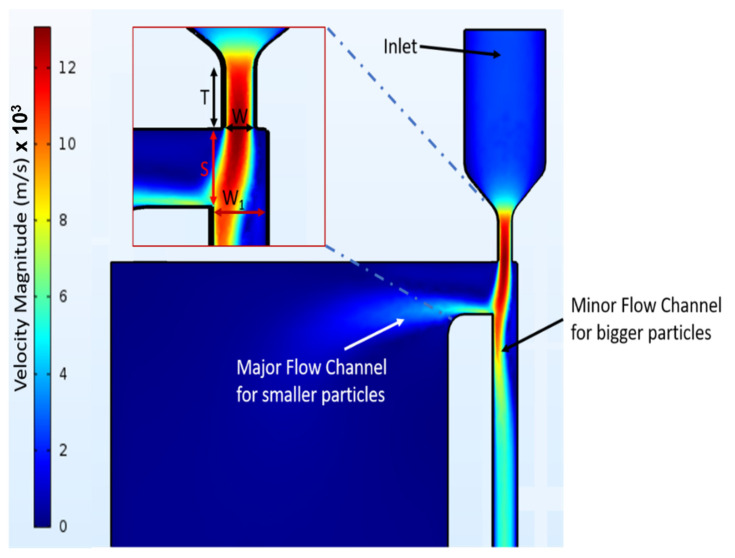
Velocity profile of the virtual impactor from Multiphysics simulations.

Aiming to minimize the size of the complete solution of the particulate matter sensor, we designed a virtual impactor with only one major flow channel. The design and the finite element simulations of the operation of the virtual impactor were carried using the COMSOL Multiphysics software. The physics modules selected for the simulations included fluid mechanics, laminar flow, and the particle-tracing module. Using the geometrical parameters and the flow rate calculated using Equations (1)–(4), the resulting geometrical model is shown in the inset of Figure 4 and in Table 1.

Figure 4 shows the simulated velocity profile of air created inside the micro channels by the fan. It is evident that the velocity is higher in the minor flow channel, compared to the major flow channel. This results in larger particles flowing into the minor flow channel because of their higher inertia and smaller (and lighter) particles being segregated into the major flow channel. The resonating sensor is placed in the major flow channel to measure the amount of the segregated particles. 

**Figure 5:**
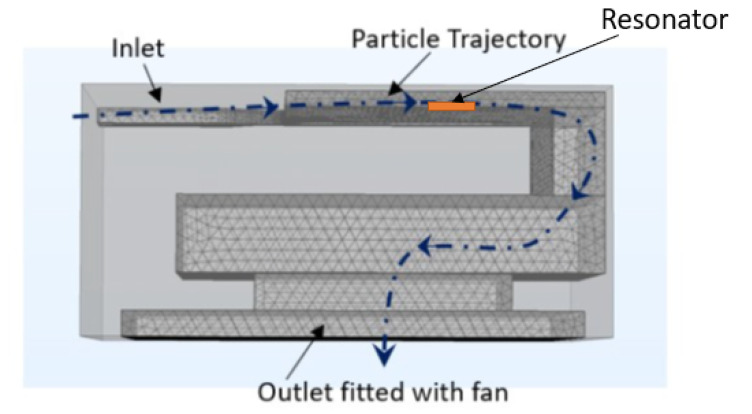
Trajectory of a particle from the inlet to the exit.

**Figure 6:**
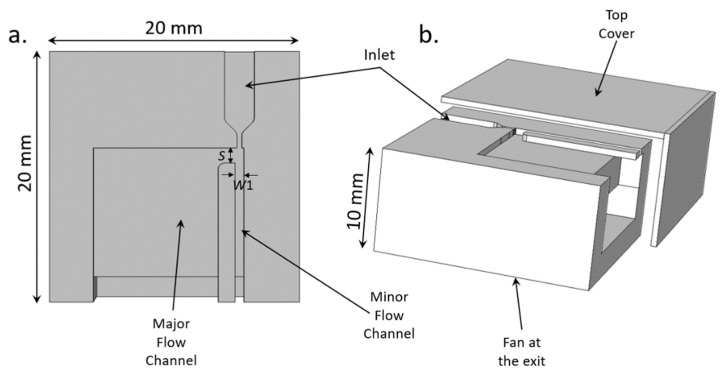
Dimensions of the complete system. (**a**) Top view, showing the minor and the major flow channels. (**b**) 3D schematic with the top cover removed to show the microchannels and the location of the external fan.

Figure 5 shows the side view of the complete system. The outer light grey block represents the 3D-printed virtual impactor package and the meshing represents the cavity inside the design (i.e., the micro-channels) through which the particles flow. The orange rectangle in the figure represents the resonator. The dashed line shows the trajectory of a particle entering through the inlet. The different grey levels inside the meshing are a result of the shadows from the line of sight in the COMSOL geometry viewer and carry no significance. The particle is segregated based on its size and enters either the minor or major flow channel. Both minor and major flow channels are connected to the exit (Figure 6a,b) through which the particle leaves the system. The flow required to maintain the operation of the virtual impactor is provided by a fan fixed at the exit. 

The overall dimensions of the system are shown in Figure 6. The dimensions of the fan are 17 mm × 17 mm × 3 mm. The system has a slot at the bottom to fit the fan, and that slot is connected to the micro-channels inside the system.

**Figure 7:**
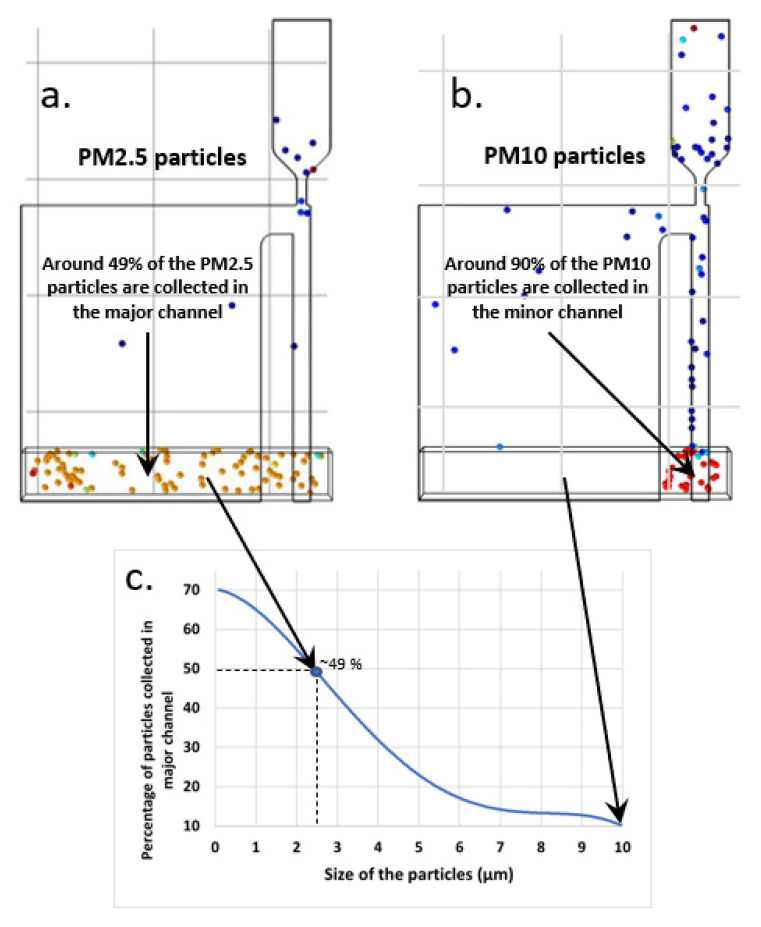
COMSOL simulation results for collection efficiency of (**a**) PM2.5 and (**b**) PM10 particles. (**a**) Shows that around 49% of the PM2.5 particles are collected in the major channel and (**b**) depicts that only about 10% of the PM10 particles are collected in the minor channel. (**c**) presents the collection efficiency of the virtual impactor for a range of particle sizes. The figure shows the percentage of the total particles collected in the major channel of the virtual impactor.

Figure 7 shows the simulation results of the collection efficiency of the designed virtual impactor. Figure 7a,b shows the collection of particles of size 2.5 microns and 10 microns in the minor and major channels of the virtual impactor. Since the virtual impactor is designed for a cut-off diameter of 2.5 microns, we observe that the collection efficiency is around 49% (intended 50%) for PM2.5 in the major channel, where the resonator will be placed. Additionally, using the same virtual impactor for particles with size of 10 microns, lowers the collection efficiency to around 10% in the major channel. We ran the simulations for particles with sizes 0.1 µm, 1 µm, 2.5 µm, 5 µm and 10 µm. Figure 7c depicts the collection efficiency for the simulated range of particle sizes in the major channels. We observe that the designed virtual impactor is quite efficient for separating particles with a size less than 2.5 µm and particles with size greater than 2.5 µm.

### 3.3. Fabrication

**Figure 8:**
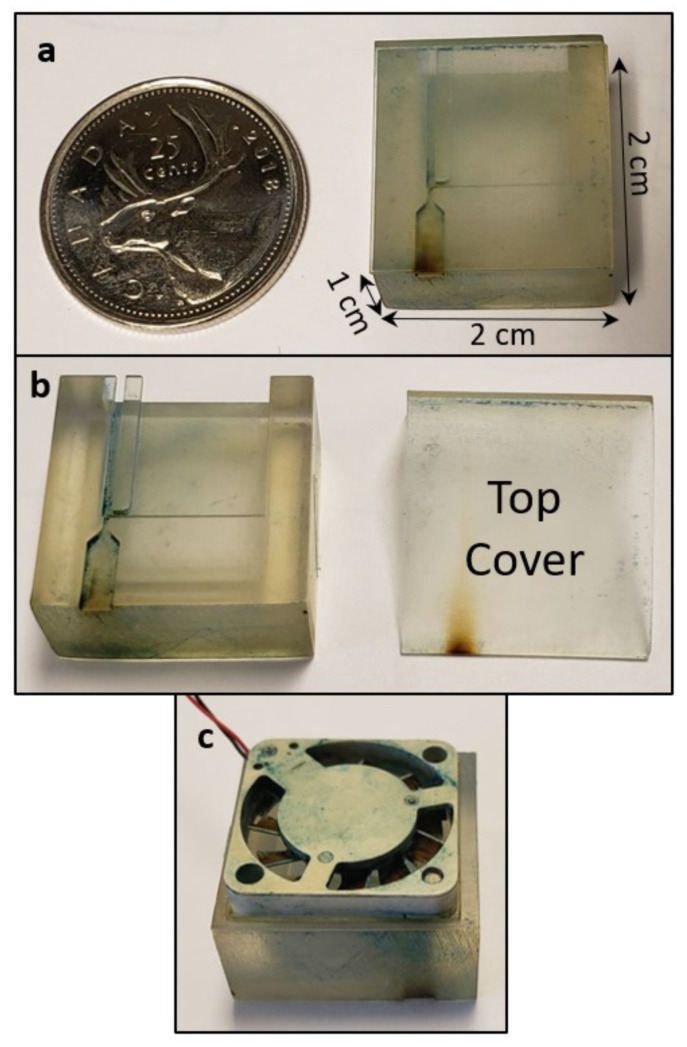
(**a**) Dimensions of the 3D-fabricated virtual impactor with the top cover, compared to a Canadian 25 cent coin. (**b**) Virtual impactor, with the top cover removed. (**c**) Fan at the outlet of the virtual impactor.

The simulated design of the virtual impactor was fabricated using 3D printing. The virtual impactor is made of semi-clear resin, as the material is easy to 3D print and is affordable. The clear resin is a semi-clear material that is convenient for micro-fluidics designs and for any parts that require translucency to see the internal features [23]. The microchannels in the virtual impactor are fabricated with a layer thickness of 50 microns using a stereolithography technology. The virtual impactor is a 3D-printed layer-by-layer using photochemical process where polymers are formed when light results in the cross-linking of monomers and oligomers (Figure 8). 

## 4. Experimental Results

**Figure 9:**
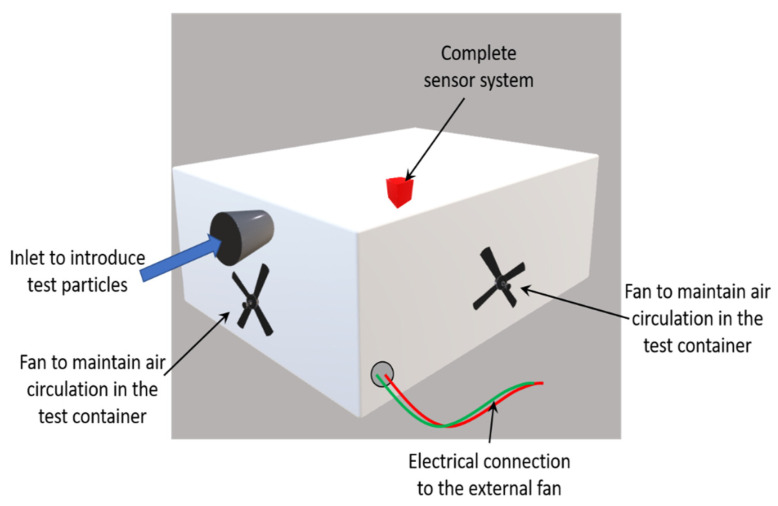
Diagram depicting the test setup with an inlet to introduce the test particles (chalk powder and fluorescent particles). The fans on the inside walls help maintain the circulation of the air inside the container. The complete sensor system is placed inside the container, with a hole that facilitates the electrical connections for the fan of the sensor.

The testing setup is shown in Figure 9. The experiment was conducted in a 51-L plastic container. Two holes were drilled on the side walls of the container. One acted as an inlet for the testing particles and the other for the electrical connections required to power the fan at the exit of the virtual impactor. 

**Figure 10:**
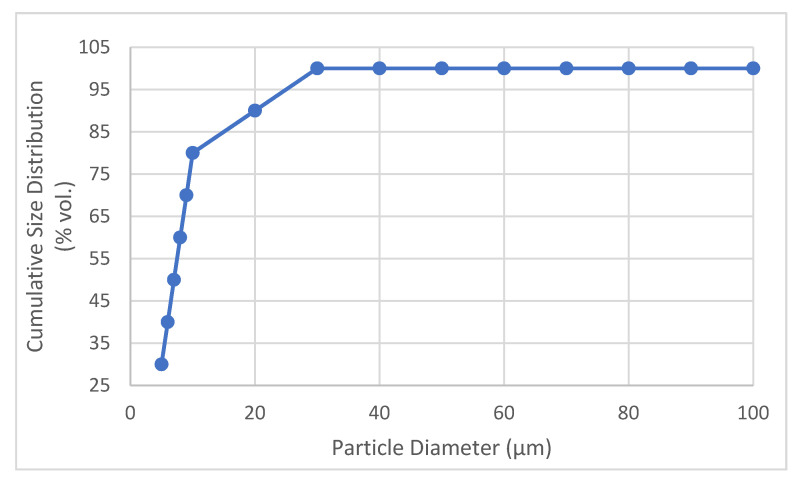
Size range of chalk powder particles.

With the virtual impactor placed carefully on the floor of the testing container, particles were introduced from the inlet hole of the container to test the particle separation capability of the impactor. Since the virtual impactor is designed for PM2.5 detection, two iterations of the test were conducted: the first was with chalk powder with particle sizes greater than 10 microns. Chalk powder is known to have particles in the size range of 5 µm to 100 µm [24]. Figure 10 shows the size range distribution of the particles in the chalk powder. Although, the particle sizes range from 5 to 100 µm, the majority of the particles (nearly 80%) are under 10 µm in size. Hence, chalk powder was considered here as PM10. The second test included a fluorescent powder with a mean particle size of 2.5 microns. The fan of the virtual impactor was turned on before introducing the particles into the testing container. The walls of the container were fitted with four small fans to maintain proper circulation of the air inside the container.

**Figure 11:**
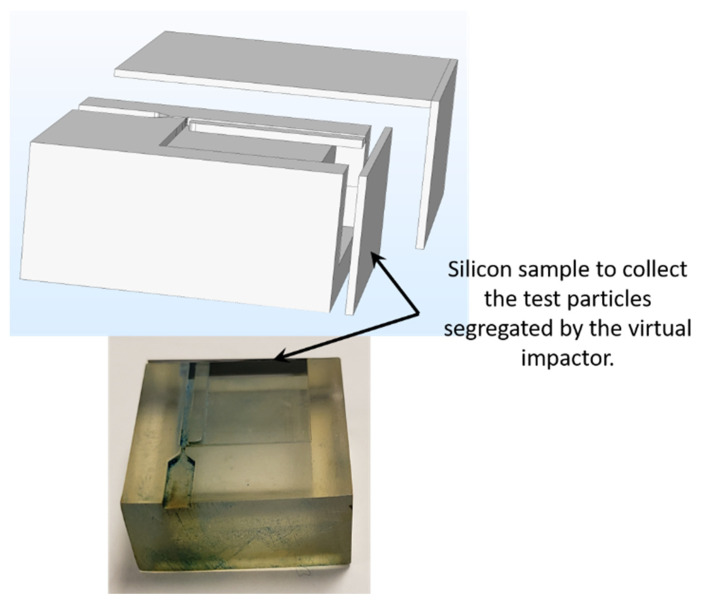
A silicon sample placed at the end of the major and minor flow channels collects the segregated particles.

**Figure 12:**
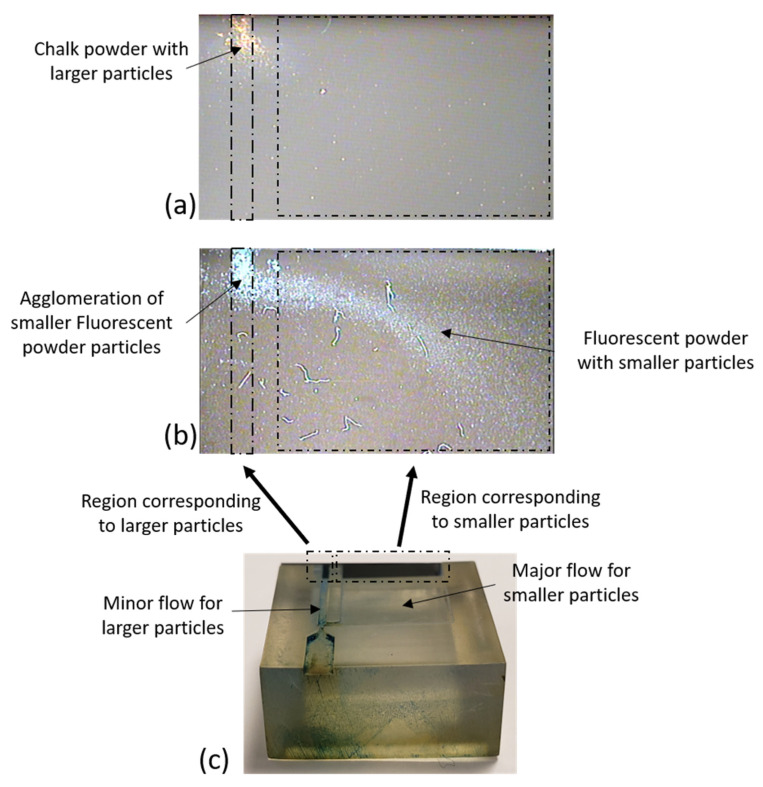
(**a**) Chalk powder particles collected in the region corresponding to the larger particles. (**b**) fluorescent powder collected in the region corresponding to the smaller particles. (**c**) Silicon sample fitted at the end of the flow channels marked with regions corresponding to the minor and the major flows.

Since the virtual impactor does not include any trap to collect particles, the particles entering the virtual impactor flowed through the microchannels and exited through the outlet of the virtual impactor (as shown in Figure 5). To test the segregation capabilities of the impactor we placed a silicon sample (carefully cut to the proper size from a bare silicon wafer) at the end of the minor and major flow channels, as shown in the Figure 11. A fraction of the particles passing through either one of the microchannels became attached to the silicon sample, which was later observed under a microscope, while the remainder of the particles exited through the outlet. A proper functioning virtual impactor should let the chalk powder particles pass through the minor flow channel because the sizes of the particles are greater than the virtual impactor’s cut-off diameter. The virtual impactor should also let the fluorescent particles pass through the major flow channel, because the particle sizes are smaller than the cut-off diameter. Figure 12 summarizes the observed experimental results.

The silicon samples were observed under a microscope, and the results confirm that the virtual impactor is successful in segregating the particles based on their sizes. Figure 12 shows that most of the chalk particles were in the region corresponding to larger particles. Additionally, the majority of the fluorescent powder particles were collected in the region corresponding to the smaller particles. There were few fluorescent powder particles observed in the region for bigger particles. This can be attributed to the fact that the fluorescent powder is vulnerable to moisture, resulting in the formation of agglomerations of the particles. These agglomerations act as larger particles and are collected in the region designed for such larger particles, further strengthening the fact that the virtual impactor is working as expected, namely, to segregate the particles based on their sizes. However, the interference of humidity can be mitigated by incorporating a high-accuracy humidity sensor with the PM sensor. By measuring the ambient humidity, we can then employ machine learning techniques to estimate the anticipated effects of humidity on the PM measurements, and therefore compensate for them computationally.

Figure 12c also shows some adsorption of particles on the walls of the channels. This issue arises mainly because of the surface roughness of the resin used in 3D printing. This can be mitigated by using injection molding techniques for fabricating the virtual impactor package, with a smooth textured finish.

## 5. Discussion

To summarize, we report the progress towards realizing one of the world’s smallest gravimetric-based particulate matter sensor. The miniaturized virtual impactor presented here is based on a novel folded design, which reduces the volume of the system by half. This paper reports the design and working principles of the virtual impactor, encapsulated in a system measuring 20 mm × 20 mm × 10 mm. Future work includes implementing the thermophoresis mechanism, in order to be able to reset the sensor of any residual particles that may deposit on it during one or more measurements. This work will eventually result in a highly compact, low-cost, and easy-to-use particulate-matter-monitoring system, without compromising the accuracy of measurements. 

## Figures and Tables

**Table 1:** Design parameters of the virtual impactor.

Design Parameter	Dimension
*W* (inlet width)	400 µm
*W*_1_ (minor flow channel width)	720 µm
*T* (inlet length)	1200 µm
*S* (major flow channel width)	840 µm
Depth of the channels	600 µm
Flow rate * (at the outlet)	0.1 m/s

* Flow rate is maintained using a fan.
